# *Pneumocystis jirovecii*-associated immune reconstitution inflammatory syndrome-like phenomenon in a child with leukaemia: a case report and literature review

**DOI:** 10.1186/s12887-022-03441-9

**Published:** 2022-07-12

**Authors:** Jia-ying Lei, Han Chen, Dun-hua Zhou, Lu-hong Xu, Jian-pei Fang, You-gang Mai

**Affiliations:** 1 Key Laboratory of Malignant Tumor Gene Regulation and Target Therapy of Guangdong Higher Education Institutes, Sun Yat-Sen University, Guangzhou, People’s Republic of China; 2 Children’s Medical Center, Sun Yat-Sen Memorial Hospital of Sun Yat-Sen University, Guangzhou, 510120 People’s Republic of China

**Keywords:** Immune reconstitution inflammatory syndrome-like phenomenon, Acute lymphocytic leukaemia, Pneumocystis Jirovecii pneumonia

## Abstract

**Background:**

Immune reconstitution inflammatory syndrome (IRIS) refers to the phenomenon of intense immune responses against pathogens in patients with AIDS undergoing antiretroviral therapy to reconstitute immune function, resulting in functional impairment of multiple organs. Non-AIDS immunosuppressed hosts may also develop similar manifestations to IRIS during immune recovery.

**Case presentation:**

An 8-year-old girl presented with acute lymphoblastic leukaemia was admitted for scheduled chemotherapy treatment. During chemotherapy, she experienced pancytopenia and Pneumocystis jirovecii pneumonia, which was diagnosed based on the abnormal shadows observed on chest computed tomography, the elevation of serum β-D-glucan, and the positive mNGS results of Pneumocystis jirovecii in both sputum and blood. After treatment with Granulocyte Colony-Stimulating Factor, sulfamethoxazole, and caspofungin, aggravation of lung lesions was discovered and severe interstitial lung disease developed in a short period along with a rapidly increasing leukocyte count. Intravenous methylprednisolone pulse therapy was given, but lung function did not improve, and she finally died after the withdrawal of medical care.

**Conclusions:**

For patients with acute lymphocytic leukaemia infected with Pneumocystis jirovecii, the rapid aggravation of pulmonary lesions in the process of blood recovery and immune reconstitution should raise vigilance against the possibility of IRIS-like reactions. The use of granulocyte stimulating factors may aggravate the inflammatory response in the lungs. The timing, dosage, and duration of treatment of glucocorticoids and the impact of high-dose methylprednisolone pulse therapy on the prognosis of patients should be explored in further research.

## Background

Pneumocystis jirovecii pneumonia is a severe opportunistic infection commonly seen in immunodeficient patients. The recovery of immune function is vital for the clearance of infections. In clinical practice, however, researchers have observed a unique phenomenon that HIV-patient with Pneumocystis jirovecii infection may experience an excessive inflammatory response which leads to further exacerbation after receiving potent anti-pneumocystis therapy and the rebuild of immune function. This phenomenon is called "immune reconstitution inflammatory syndrome (IRIS)" [1].

IRIS refers to developing an excessive immune response against pathogens in patients with AIDS undergoing antiretroviral therapy, resulting in functional impairment of multiple organs [2]. IRIS is associated with both adaptive and innate immunity of the organism against the pathogen [3]. Pneumocystis jirovecii is one of the most common pathogens causing IRIS. Approximately 5% of patients with AIDS who were infected with Pneumocystis jirovecii developed IRIS. Because the pulmonary was the most commonly involved organ, about half of these patients may experience respiratory failure [1, 2]. Non-AIDS immunosuppressed hosts may also show similar manifestations of IRIS during immune recovery [4].

IRIS-like phenomena can occur in non-AIDS immunosuppressed hosts such as patients with connective tissue disease and organ transplant patients receiving immunosuppressive therapy [5]. But the data is rare, especially in the pediatric patient. Here, we reported a case of an IRIS-like phenomenon in a child with acute lymphoblastic leukaemia infected with Pneumocystis jirovecii during post-chemotherapy leukocyte recovery and reviewed the relevant literature, aimed to heighten the clinician's vigilance to this syndrome and give some suggestions.

## Case presentation

An 8-year-old female presented to the pediatric haematology-oncology department at the Sun Yat-sen Memorial Hospital for scheduled chemotherapy treatment. The child was diagnosed with acute lymphoblastic leukaemia (Pre-B-ALL, BCR/ABL p190 positive) 2 months ago and the risk stratification was set as high risk.

**Fig. 1:**
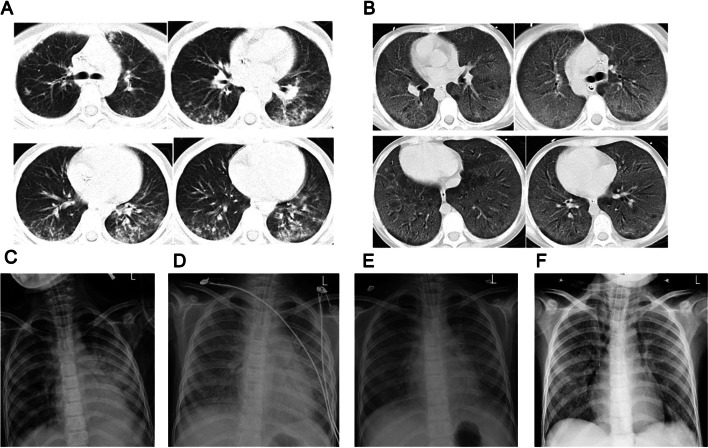
Chest CT and chest X-ray in the clinical course of the patient: **A** CT on the 4^th^ day of fever showed multiple small patchy hyperdense shadows in both lungs with poorly defined borders, mainly in the lower lobes of both lungs. **B** CT on the 16^th^ day of fever showed the appearance of interstitial thickening with grid-like changes and ground glass opacities in both lungs. **C** A chest X-ray showed large dense shadows in both the middle and lower lung (7^th^ day of fever). **D** A chest X-ray showed the increase of large dense shadows of pulmonary infiltrates (9^th^ day of fever). **E** A chest X-ray showed an increase in grid-like changes and corn-like high-density shadows in both lungs with unclear borders (11^th^ day of fever). **F** A chest X-ray showed no change in grid-like changes and corn-like high-density shadows

The patient commenced the first round of CAM (cyclophosphamide + cytarabine + 6-mercaptopurine) regimen of induction chemotherapy along with oral dasatinib treatment. The child experienced neutropenia and developed a fever (38.5 °C) with occasional cough and pharyngeal discomfort. Chemotherapy was stopped and meropenem was used. Fever repeated with elevated fever peaks. C-reactive protein (CRP) increased to 23.3 mg/dL, while blood and sputum cultures were negative. Computed tomography (CT) scan showed multiple small patchy hyperdense shadows in both lungs with poorly defined borders, mainly in the lower lobes of both lungs (Fig. 1A). According to the examination results and the agranulocytosis state, teicoplanin combined with trimethoprim-sulfamethoxazole(TMP-SMX) and caspofungin were prescribed. Methylprednisolone (1 mg/kg qd) was also utilised. The patient's temperature did not decrease, and dyspnea gradually developed. The NGS test result indicated a Pneumocystis jirovecii infection(Table 1), and ticoranine was discontinued.

**Table 1:** NGS report of the microorganism in sputum

Blood		
Genus	Species	Sequencing reads
Pneumocystis	*Pneumocystis jirovecii*	481
Hymenobacter	*Hymenobacter mucosus*	4
Sputum		
Genus	Species	Sequencing reads
Pneumocystis	*Pneumocystis jirovecii*	7407

**Fig. 2:**
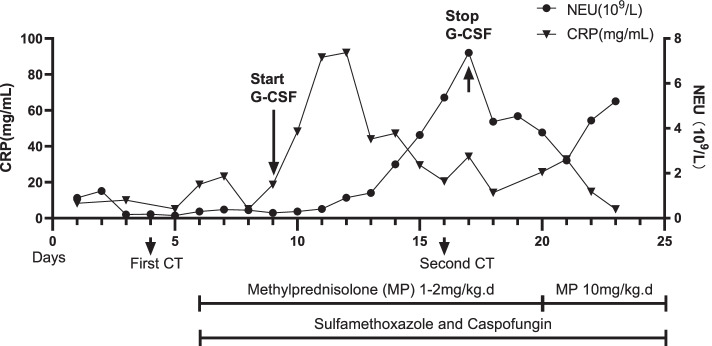
The change of neutrophil count and CRP in the clinical course of the patient

The child's dyspnea significantly worsened, and the PaO_2_ was 59 mmHg (with an oxygen supplementation via mask at 8L/min). She was transferred to the pediatric intensive care unit (PICU) and intubated. Chest X-ray showed large dense shadows in the middle and lower lungs (Figs. 1C and D). The Procalcitonin(PCT) was mildly elevated(0.65 ng/ml). The 1,3-beta-D-glucan test (G test) result was positive (634 pg/mL) while the galactomannan test (GM test) result (0.33 μg/L) was negative. EBV, CMV nucleic acid test, as well as blood and sputum cultures were negative. Considering the severity of pneumonia and neutrophil deficiency after chemotherapy, linezolid was commenced for anti-infection treatment, and granulocyte colony-stimulating factor (G-CSF) (5 ug/kg.d) was administered to promote proliferation and differentiation of the bone marrow granulocyte lineage. Subsequently, the neutrophil count and C-reactive protein (CRP) began to rise rapidly (Fig. 2).The sputum mNGS test result also showed a Pneumocystis jirovecii infection(Table 1) 2 days later, and the anti-infective regimen remained unchanged.

The patient's temperature decreased, but a gradually increasing ventilator parameter was required to maintain the oxygen saturation. The oxygenation index (OI) was between 14-and 16, implying the existence of moderate acute respiratory distress syndrome, according to the Pediatric Acute Lung Injury Consensus (PALICC). Inflammatory factor levels were significantly increased: sIL-2R (soluble IL-2 receptor, soluble IL-2 receptor) 5049 U/mL, IL-6 1141 pg/mL, IL-10 70.9 pg/mL, and IL-8 1242 pg/mL (Table 2). The chest X-ray showed multiple speckled, corn-like dense shadows in both lungs with indistinct borders and lattice-like changes (Fig. 1E), T-SPOT and serological tests of common respiratory pathogens (Mycoplasma pneumonia, adenovirus, respiratory syncytial virus, parainfluenza virus, influenza virus A, influenza virus B, Legionella type 1–7, coxsackievirus A, coxsackievirus B, echovirus IgM) were negative. Meropenem and linezolid were replaced with amikacin and tigecycline. The peak and the frequency of fever decreased, but the ventilator parameters were still high, with OI values between 10 and 13.

**Table 2:** The cytokine in the clinical course of the patient

	1^st^ day of fever	3^rd^ day of fever	8^th^ day of fever	12^th^ day of fever
sIL-2R (U/mL)	1412	1635	**5049**	3854
IL-6 (pg/mL)	47	56.9	**1141**	32.1
IL-10 (pg/mL)	< 5.00	16.2	**70.9**	23.2
TNF-α (pg/mL)	< 4.00	6.9	**10.6**	13.3
IL-8 (pg/mL)	62.8	71.1	**1242**	67.3
IL-1β (pg/mL)	< 5.00	< 5.00	< 5.00	< 5.00

On the 16^th^ day, the CT scan showed that the patchy hyper-dense shadows were alleviated, but interstitial thickening with grid-like changes and ground-glass opacities appeared in both lungs, suggestive of bilateral interstitial pneumonia (Fig. 1B). The fibrosis markers significantly increased: collagen type IV (CIV) 140.12 ng/mL, pre-collagen peptide III (PCIII) 151.18 ng/mL, and hyaluronic acid (HA) 1056.81 ng/mL. High-dose methylprednisolone (10 mg/kg.d × 5 d) pulse therapy was given, along with the anti-fibrotic treatment of pirfenidone and acetylcysteine. But the lung function did not improve, and the X-ray showed corn-like high-density shadows in both lungs with unclear borders and grid-like changes (Fig. 1E). The child's parents opted for withdrawal of medical care, and the girl died after being discharged from the hospital.

## Discussion and conclusions

Pneumocystis Jirovecii pneumonia (PCP) is a severe opportunistic infection among immunocompromised patients, not only HIV patients but also haematology patients, especially patients with ALL and allogeneic HSCT recipients [6]. In immunocompromised patients, Pneumocystis jirovecii could proliferate due to insufficient immune cell infiltration, damaging alveolar epithelial cells and affecting respiratory function [3]. Immune reconstitution combined with anti-pneumocystis therapy is critical for treating PCP. However, during PCP treatment a deathful phenomenon may appear called immune reconstitution inflammatory syndrome (IRIS).

In patients with AIDS, the diagnosis of IRIS relies on the atypical clinical manifestations of opportunistic infections, the marked decrease in HIV viral load, and the rapid increase in CD4 + cells [7]. There are no uniform diagnostic criteria for IRIS-like reactions in non-AIDS patients. Hirohiko et al. proposed brief diagnostic criteria for IRIS-like reactions in non-AIDS immunosuppressed hosts: 1) the patient is HIV negative; 2) the disease occurs during the process of immune recovery of the patient; 3) the inflammatory response is considered to be caused by a pathogen that the host was infected with before or during immune reconstitution; 4) exclusion of worsening of the primary disease and infection with new pathogens after immune reconstitution [4].

The patient in this case with acute lymphoblastic leukaemia was in an immunosuppressed state after chemotherapy treatment. The diagnosis of PCP in this child was clear according to the results of mNGS tests of peripheral blood and sputum, fungal D-glucan levels, and pulmonary imaging. In this case, a highly effective treatment (sulfamethoxazole combined with caspofungin) against PCP was started in the early stage of the disease before severe respiratory symptoms appeared, but the patient's manifestation was not relieved during the gradual recovery of the hemogram. Instead, severe interstitial lung disease developed and rapidly progressed to acute respiratory distress syndrome with markedly elevated levels of cytokines. It is highly suggested that the lung lesions may be associated with an intense immune response during immune recovery, consistent with the diagnostic criteria for an IRIS-like reaction.

Cytokine levels are of value in assessing the severity of inflammatory response in patients with PCP. It was found that the mRNA levels of IL-2, IL-4, IL-10, and IL-13 in peripheral blood were significantly higher in patients with PCP. PCP patients with an increased expression of IL-2 mRNA were more likely manifested with wheezing, and chest X-ray findings such as central perihilar infiltrate, patchy infiltrates, consolidation, hilar lymphadenopathy, pneumothorax, pleural effusion. A high-level expression of IL-10 mRNA in PCP patients was associated with weight loss, dyspnea, night sweats, wheezing, and abnormal findings of chest X-ray [8]. Previous studies also found that an imbalance of pro-inflammatory and anti-inflammatory cytokines existed in non-AIDS immunocompromised patients with severe PCP. Patients with PaO_2_/FiO_2_ < 200 mmHg had significantly higher levels of IL-8 and IL-8/IL-10 ratio in both blood and bronchoalveolar lavage fluid (BALF) than those with PaO2/FiO2 > 200 mmHg, and significantly increased levels of IL-8, IL-8/IL-10 ratio in BALF were found in the patients who required mechanical ventilation and in non-survivors [9]. Besides, it was previously reported that patients who received corticosteroids and later developed IRIS had a marked increase in IL-6, IL-8, and IFN-γat the time of IRIS, implying that increased IL-8, Th1, and Th17 cytokine levels may be helpful in identifying patients at high risk for IRIS [10]. In this case, serum soluble IL-2 receptor (sIL-2R), IL-6, IL-10 and IL-8 levels were significantly elevated during the progressive phase of the disease.

Drugs may also promote the development of IRIS. G-CSF was used in this case for immune reconstitution due to severe infection as well as neutropenia. As neutrophils rapidly rose, IL-8 significantly elevated. IL-8 is a representative of the CXC-type subfamily of chemokines, which has a chemotactic effect on neutrophils and can be produced by peripheral blood monocytes [11]. The significantly elevated serum IL-8 levels suggested a possible neutrophil-mediated inflammatory response. sIL-2R was released by activated CD4 + T cells, which suggested that CD4 + T cells also played an important role in the pathological changes in the lungs of this child [12]. Though there is no previous report showing that G-CSF can induce IRIS, in this case, we consider G-CSF as a promoter of IRIS-like reaction by increasing the number and function of neutrophils [13], which in turn enhances the neutrophil-mediated inflammatory response. Furthermore, the use of chemotherapeutic agents which had pulmonary toxicity could also lead to lung injuries, such as dasatinib and cyclophosphamide [14, 15], although they were not regarded as the main reason in this case.

Treatment of PCP-associated IRIS includes complete clearance of the pathogen and suppression of the overwhelming immune response. The use of glucocorticoids is recommended because it has been shown to reduce mortality in patients with AIDS combined with severe PCP in large samples of randomised clinical trials [16]. However, the value of glucocorticoids in non-AIDS patients with PCP remains controversial [17–19]. Investigators tend to believe that glucocorticoid application could improve the prognosis of patients with IRIS, especially those presenting with acute respiratory distress syndrome [2]. But in this case, glucocorticoids treatment was invalid. Though a regular dose of glucocorticoid was given at the early stage, the lung function still deteriorated, which was considered a result of IRIS. The CT scan result and the significantly increased levels of fibrosis markers suggested that fibrosis progress was initiated [20]. A methylprednisolone pulse therapy combined with pirfenidone and acetylcysteine was utilised, but it could not reverse the condition. However, the timing, dose, duration of glucocorticoid administration and the effect of high-dose glucocorticoid treatment on the prognosis of patients require further study.

To our knowledge, the case of developing interstitial pneumonia with fibrosis after Pneumocystis jirovecii infection in the pediatric patient with hematological malignant diseases has not been reported before. In a previously reported case, an HIV patient received a high dose of methylprednisolone and a followed regular dose of prednisolone when the respiratory function and imaging findings were not improved after anti-pneumocystis therapy and antiretroviral therapy, which was similar to the present patient. The patient was alive, but the corticosteroids treatment was deemed to elicit no positive effects upon lung fibrosis and was stopped. In the present case, the patient died [21]. Previous studies assumed that adjunctive steroid therapy was beneficial to reduce mortality and respiratory complications with PCP, but once fibrosing was initiated, corticosteroids seemed insufficient.

This article reports a case of IRIS-like reaction associated with PCP in a non-AIDS immunosuppressed pediatric patient and reviews the relevant literature. The case reveals that no matter HIV infection or not, for immunocompromised patients infected with Pneumocystis jirovecii, immune reconstitution inflammatory response syndrome may occur. The intense inflammatory response can lead to irreversible damage, and abnormally increased levels of inflammatory factors are predictive of the IRIS-like reaction diagnosis. Clinicians should be vigilant about it. Although the glucocorticoids treatment for IRIS-like reaction in non-AIDS immunosuppressed patients is still controversial, we tend to believe that an early and proper dose of glucocorticoids can help to alleviate inflammatory response and play a positive role in reducing the occurrence of this syndrome.

## Data Availability

The datasets generated during and/or analysed during the current study are available from the corresponding author on reasonable request.

## Abbreviations

AIDS: Acquired immune deficiency syndrome
ALL: Acute lymphocytic leukemia
CAM: Cyclophosphamide + Cytarabine + 6-mercaptopurine
CMV: Cytomegalovirus
CIV: Collagen type IV
EBV: Epstein-Barr virus
G-CSF: Granulocyte Colony-Stimulating Factor
GM test: Galactomannan test
G test: 1,3-Beta-D-glucan test
HA: Hyaluronic acid
HIV: Human immunodeficiency virus
HSCT: Hematopoietic stem cell transplantation
IFN-γ: Interferon gamma
IL: Interleukin
IRIS: Immune reconstitution inflammatory syndrome
mNGS: Metagenomics next generation sequencing
OI: Oxygenation index
PALICC: Pediatric Acute Lung Injury Consensus
PCIII: Pre-collagen peptide III
PCP: Pneumocystis Jirovecii pneumonia
PCT: Procalcitonin
T-SPOT: T cell enzyme-linked immunospot
VDLD: Dexamethasone + Vincristine + Daunorubicin + Pegaspargase
